# ICT-based adherence monitoring in kidney transplant recipients: a randomized controlled trial

**DOI:** 10.1186/s12911-020-01146-6

**Published:** 2020-06-10

**Authors:** Hee-Yeon Jung, Yena Jeon, Sook Jin Seong, Jung Ju Seo, Ji-Young Choi, Jang-Hee Cho, Sun-Hee Park, Chan-Duck Kim, Young-Ran Yoon, Se-Hee Yoon, Jong Soo Lee, Yong-Lim Kim

**Affiliations:** 1 Division of Nephrology, Department of Internal Medicine, School of Medicine, Kyungpook National University, Kyungpook National University Hospital, Daegu, 41944 South Korea; 2 Department of Statistics, Kyungpook National University, Daegu, South Korea; 3 Department of Biomedical Science and Clinical Trial Center, Kyungpook National University, Kyungpook National University Hospital, Daegu, South Korea; 4 Department of Internal Medicine, Konyang University College of Medicine, Daejeon, South Korea; 5 Department of Internal Medicine, University of Ulsan College of Medicine, Ulsan, South Korea

**Keywords:** Information and communication technology, Feedback, Adherence, Kidney transplantation

## Abstract

**Background:**

Prior studies have explored the use of regular reminders to improve adherence among kidney transplant recipients (KTRs), but none have included real-time alarms about drug dosage, frequency, and interval. In the present study, we aimed to evaluate the efficacy and stability of an information and communication technology (ICT)-based centralized monitoring system for increasing medication adherence among Korean KTRs.

**Methods:**

In this prospective, multicenter, randomized controlled study, enrolled KTRs were randomized to either the ICT-based centralized monitoring group or control group. The ICT-based centralized monitoring system alerted both patients and medical staff with texts and pill box alarms if there was a missed dose or a dosage/time error. We compared the two groups in terms of medication adherence and transplant outcomes over 6 months, and evaluated patient satisfaction with the ICT-based monitoring system.

**Results:**

Among 114 enrolled KTRs, 57 were assigned to the ICT-based centralized monitoring group and 57 to the control group. The two groups did not significantly differ in mean adherence at each follow-up visit. The intrapatient variability of tacrolimus and mycophenolic acid levels, renal function, and adverse transplant outcomes did not differ between the intervention and control groups, or between the intervention group with feedback generation and the intervention group without feedback generation. Patients showed high overall satisfaction with the ICT-based centralized monitoring system, which significantly improved across the study period (*p* = 0.012).

**Conclusions:**

Due to high baseline adherence, the ICT-based centralized monitoring system did not maximize medication adherence or enhance transplant outcomes among Korean KTRs. However, patients were highly satisfied with the system. Our results suggest that the ICT-based centralized monitoring system could be successfully applied in clinical trials.

**Trial registration:**

ClinicalTrials.gov, NCT03136588. Registered 20 April 2017 - Retrospectively registered.

## Background

Among kidney transplant recipients (KTRs), non-adherence to immunosuppressive medications is a major cause of antibody-mediated rejection, which leads to graft loss [1, 2]. Immunosuppressant non-adherence also contributes to the increased healthcare costs [3] associated with acute rejection treatment, additional hospitalization, and re-dialysis. Therefore, it is crucial to monitor KTRs who require long-term pharmacotherapy, and prevent non-adherence.

Previous studies have reported that approximately 14–36% KTRs are non-adherent to immunosuppressive medications [4–6]. Medication non-adherence can be either intentional or unintentional. We hypothesized that unintentional forgetfulness regarding immunosuppressive medications in KTRs could be improved by continuous electronic monitoring of adherence, and by providing patients with reminder alarms from the information and communication technology (ICT)-based centralized monitoring system. Previous studies have attempted to improve medication adherence among KTRs by using technology-based adherence-promoting interventions [7–9]. Compared to prior investigations, the main methodologic difference in our present study is that we provided real-time alarms about both drug dose and interval, and used a smart pill box to determine adherence, including dose-taking adherence, dose-frequency adherence, frequency, and dose-interval adherence.

In the present study, we aimed to determine the efficacy and stability of an ICT-based centralized monitoring system with regards to improving adherence to immunosuppressive medication and transplant outcomes in KTRs.

## Methods

### Study overview

Details about the study protocol have been previously described [10]. Briefly, 114 KTRs who completed the informed consent form were registered and randomly assigned in a 1:1 ratio to the ICT-based centralized clinical trial monitoring group or the ambulatory follow-up group. The planned follow-up duration was 6 months. After randomization, both groups were scheduled to for 6 visits: at 4, 8, 12, 16, 20, and 24 weeks. In the ICT-based centralized clinical trial monitoring group, both patients and the medical staff received feedback in the form of texts and pill box alarms in the event of a dosage/dosing time error or a missed dose.

### Feedback algorithms

In the ICT-based centralized monitoring group, both participants and medical staff received feedback in the form of text message alarms regarding missed doses, misuse, or overuse of the medication. In the event of a missed immunosuppressant dose, the first violation generates feedback within 1 h at the break of the ±3 h range from the fixed dosing time. If the dose is still not taken after the feedback, up to two additional texts are sent at a 30-min interval. Feedback was also sent within 1 h from the moment of recognition in the event of any discrepancy between the dosage taken and the dosage prescribed, and if a dose was taken outside of the allowed ±3 h dosing time range.

### Hypothesis and limitations

We hypothesized that patients failed to take their medications due to unintentional forgetfulness, and that the ICT-based centralized monitoring system could improve medication adherence among KTRs. Notably, this system does not include a camera to record patients at the moment of medication ingestion and, therefore, cannot improve intentional non-adherence.

### Patient selection

The patient inclusion criteria were as follows: age of ≥8 years; underwent kidney transplantation ≥1 month ago; maintained stable renal function after kidney transplantation, with an estimated glomerular filtration rate (eGFR) of ≥30 mL/min/1.73 m^2^; history of kidney transplantation only, with no other organ transplantations; use of tacrolimus, mycophenolic acid, and steroids for post-transplant immunosuppression; signed the informed consent form in compliance with due process; and capable of making office visits and participating in the trial in accordance with the protocol.

The patient exclusion criteria were as follows: refusal of the ICT-based centralized home monitoring; history of treatment for acute rejection within the past 3 months; active infectious disease; uncorrected ischemic heart disease; visual or auditory impairments that could affect use of the smart pill box; inability to provide fingerprint authentication of personal identity (e.g., due to adermatoglyphia); illiteracy; lack of smartphone and unable to receive text messages; and other investigator-determined reasons that made participation in the clinical trial inappropriate.

### Primary outcomes

The primary outcome was medication adherence. Dose-taking adherence, dose-frequency adherence, dose-interval adherence, and drug holidays were assessed based on the smart pill box data in the ICT-based centralized monitoring group, and based on the drug administration diary in the ambulatory follow-up group. Dose-taking adherence was calculated as (the number of pills taken over a certain time period/the number of pills prescribed over the same period) × 100%. Dose-frequency adherence was calculated as (the number of days of correct daily dosing over a certain time period/the number of days in the same period) × 100%. Dose-interval adherence was calculated as (the number of correct dosing intervals over a certain time period/the number of days in the same period) × 100%. A correct dosing interval was defined with a margin of ±25%. In this trial, the medication was to be taken twice daily, with a 12-h dosing interval. Thus, the allowed dosing interval ranged from 9 to 15 h. A drug holidays was calculated as (the number of days without taking the medication/the number of days in the same period) × 100%.

### Secondary outcomes

The secondary outcomes included transplant outcomes and patient satisfaction with the system. Trough levels of tacrolimus and mycophenolic acid, coefficient of variation (CV) of drug levels, and eGFR were measured at 4, 8, 12, 16, 20, and 24 weeks of follow-up. To determine within-patient variability in immunosuppressant trough levels, the CV (%) was calculated as (the standard deviation/mean trough level of the immunosuppressant) × 100 [11]. Panel-reactive antibody (PRA) was assessed at baseline and at 24 weeks. At 12 weeks, we measured serum BK virus, which is a well-known etiologic agent of viral infection in KTRs [12]. To evaluate clinical outcome, we analyzed the development of biopsy-proven acute rejection (BPAR) and death-censored graft loss (DCGL) during follow-up. At 4 and 24 weeks, a patient satisfaction questionnaire was administered in the ICT-based centralized clinical trial monitoring group to determine whether the patients were satisfied with the system. This questionnaire is presented in additional file 1. This questionnaire was conducted because patient reported outcomes, including satisfaction with the system, are important, and it is important to ensure that patients are willing to use the system in the future.

### Statistical analysis

Between-group differences were tested by independent sample *t*-tests and chi-squared tests, as appropriate. The inter-group difference in immunosuppressant adherence was assessed using the *t*-test, and intra-group variation was analyzed with the paired *t*-test. Statistical analyses were performed using the SAS system for Windows, version 9.2 (SAS Institute Inc., Cary, NC). A *p* value of < 0.05 was considered statistically significant.

## Results

### Study participants

**Fig. 1:**
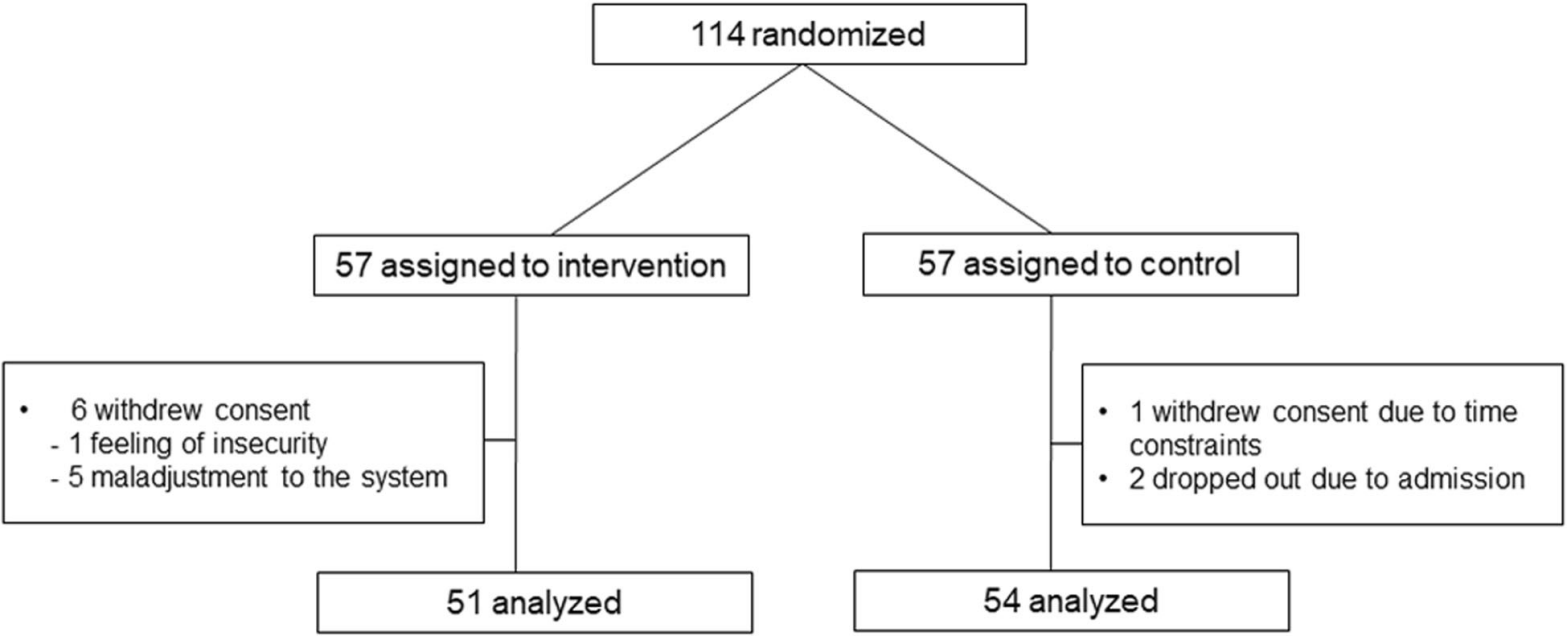
Flow of participants inclusion. A total of 114 KTRs were randomized 1:1 into the intervention group (*n* = 57) or control group (*n* = 57). After excluding patients who withdrew consent or dropped out, the final analyses included 51 KTRs in the intervention group and 54 in the control group

Figure 1 shows patient inclusion in a flowchart. A total of 114 KTRs were randomized 1:1 into the intervention group (*n* = 57) or control group (*n* = 57). After excluding patients who withdrew consent or dropped out, the final analyses included 51 KTRs in the intervention group and 54 in the control group. Table 1 shows the baseline characteristics of the included patients. The mean age was 49.9 years in the intervention group, and 49.0 years in the control group. Males comprised 60.8% of the intervention group, and 53.7% of the control group. Living donor KT had been performed in 47.1% of the intervention group, and 35.2% of the control group. The mean eGFR was 69.7 in the intervention group, and 74.3 in the control group.

**Table 1:** Baseline characteristics

	Intervention (*n* = 51)	Control (*n* = 54)
Age, years	49.9 ± 10.0	49.0 ± 12.2
Male, *n* (%)	31 (60. 8)	29 (53.7)
Smoking, *n* (%)		
Non-smoker	39 (76.5)	48 (88.9)
Ex-smoker	8 (15.7)	6 (11.1)
Current smoker	4 (7.8)	0 (0)
Time after KT, months	30.7 ± 19.8	15.7 ± 9.5
Primary renal disease, *n* (%)		
Diabetes	13 (25.5)	13 (24.1)
Non-diabetes	38 (74.5)	41 (75.9)
Donor age, years	46.0 ± 12.6	45.3 ± 14.8
Donor male, *n* (%)	25 (49.0)	31 (59.6)
Donor type, *n* (%)		
Living	24 (47.1)	19 (35.2)
Deceased	27 (52.9)	35 (64.8)
Number of HLA mismatch		
Total	3.5 ± 1.9	3.0 ± 1.6
DR	1.1 ± 0.8	1.0 ± 0.6
PRA > 10%, *n* (%)	11 (21.6)	13 (24.1)
Baseline laboratory data		
Creatinine, mg/dL	1.1 ± 0.4	1.1 ± 0.3
eGFR, mL/min/1.73 m^2^	69.7 ± 19.0	74.3 ± 22.2

Values are shown as mean ± standard deviation or number (%)

*eGFR* estimated glomerular filtration rate, *HLA* Human leukocyte antigen, *KT* Kidney transplantation, *PRA* Panel-reactive antibody

### Adherence

**Fig. 2:**
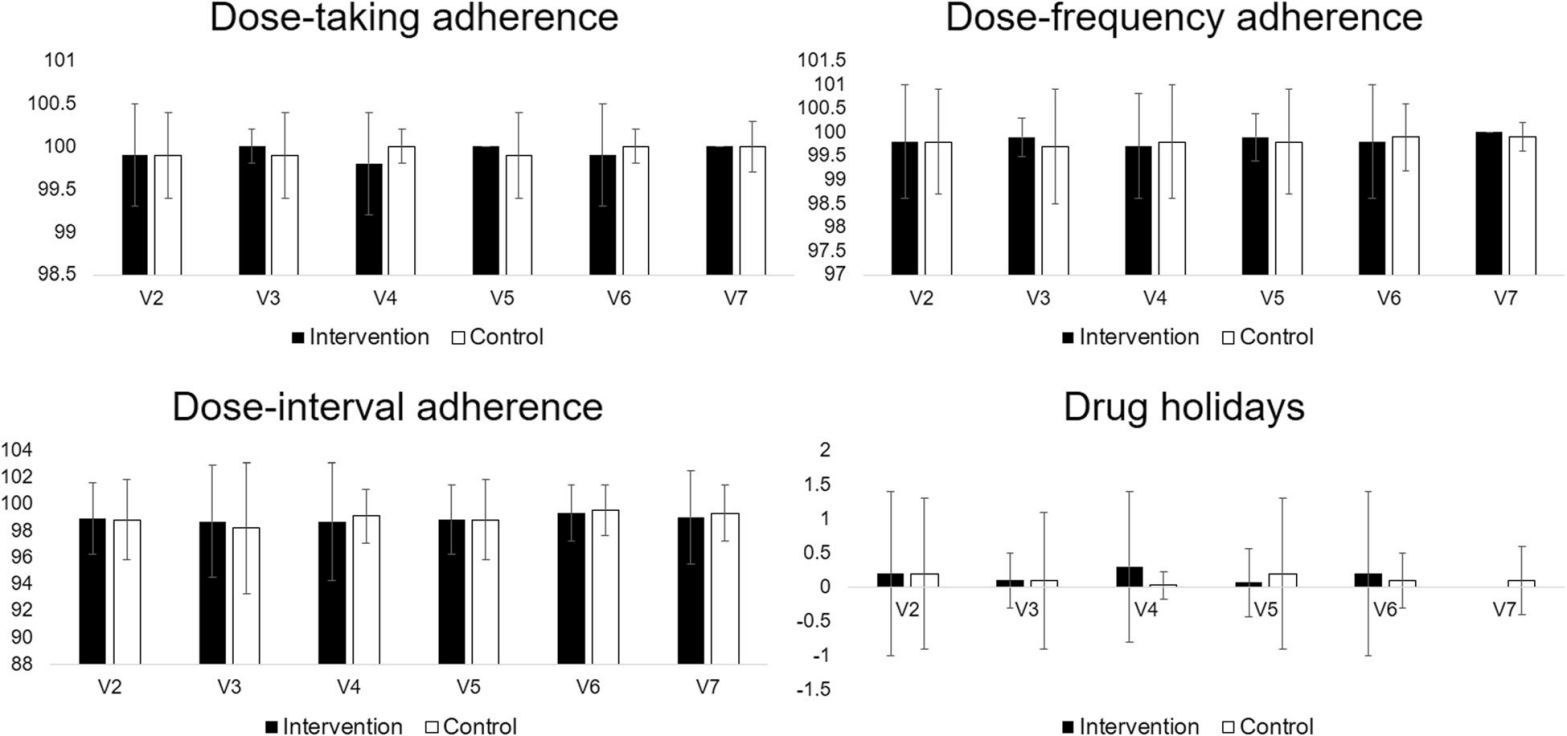
Dose-taking adherence, dose-frequency adherence, dose-interval adherence, and drug holidays at each period. The two patient groups did not significantly differ in adherence in terms of dosing, time, or drug holidays

Figure 2 shows dose-taking adherence, dose-frequency adherence, dose-interval adherence, and drug holidays at each period. Patients in both groups had > 98% adherence throughout the entire study period. The two groups did not significantly differ in adherence, including dosing, time, and drug holidays.

### Transplant outcomes between the intervention and control groups

Table 2 presents transplant outcomes. The intervention and control groups did not significantly differ in the tacrolimus trough levels (5.3 ± 1.2 vs. 5.0 ± 1.2, *p* = 0.282), tacrolimus CV (23.9 ± 13.5 vs. 25.1 ± 11.4, *p* = 0.645), mycophenolic acid trough levels (2.8 ± 1.6 vs. 2.6 ± 1.3, *p* = 0.600), mycophenolic acid CV (37.9 ± 17.3 vs. 38.9 ± 19.4, *p* = 0.783), or renal allograft function at 4 weeks (67.8 ± 18.2 vs. 71.4 ± 21.8, *p* = 0.365) or at 24 weeks (65.2 ± 18.9 vs. 70.2 ± 21.0, *p* = 0.203). Moreover, there was no significant between-group difference in the incidence of development of de novo anti-HLA antibodies (5.9% vs. 14.8%, *p* = 0.135). Neither BPAR nor DCGL occurred.

**Table 2:** Transplant outcomes in the intervention and control groups

	Intervention (*n* = 51)	Control (*n* = 54)	*p* value
Drug levels			
TAC trough level, ng/mL	5.3 ± 1.2	5.0 ± 1.2	0.282
TAC CV^a^	23.9 ± 13.5	25.1 ± 11.4	0.645
MPA trough level, μg/mL	2.8 ± 1.6	2.6 ± 1.3	0.600
MPA CV^a^	37.9 ± 17.3	38.9 ± 19.4	0.783
eGFR			
4 weeks	67.8 ± 18.2	71.4 ± 21.8	0.365
8 weeks	67.9 ± 19.7	71.3 ± 19.2	0.373
12 weeks	66.7 ± 19.4	71.3 ± 21.6	0.262
16 weeks	67.6 ± 17.4	72.4 ± 21.9	0.213
20 weeks	66.3 ± 18.0	71.6 ± 21.8	0.182
24 weeks	65.2 ± 18.9	70.2 ± 21.0	0.203
Number of events, *n* (%)			
De novo anti-HLA antibodies	3 (5.9)	8 (14.8)	0.135
BK viremia	1 (2.0)	1 (1.9)	1.000
BPAR	–	–	
DCGL	–	–	

Values are shown as mean ± standard deviation or number (%)

*BPAR* Biopsy-proven acute rejection, *CV* Coefficient of variation, *DCGL* Death-censored graft loss, *eGFR* estimated glomerular filtration rate, *HLA* Human leukocyte antigen, *MPA* Mycophenolic acid, *TAC* Tacrolimus

^a^CV = (standard deviation/mean) × 100%

### Transplant outcomes according to feedback generation

**Fig. 3:**
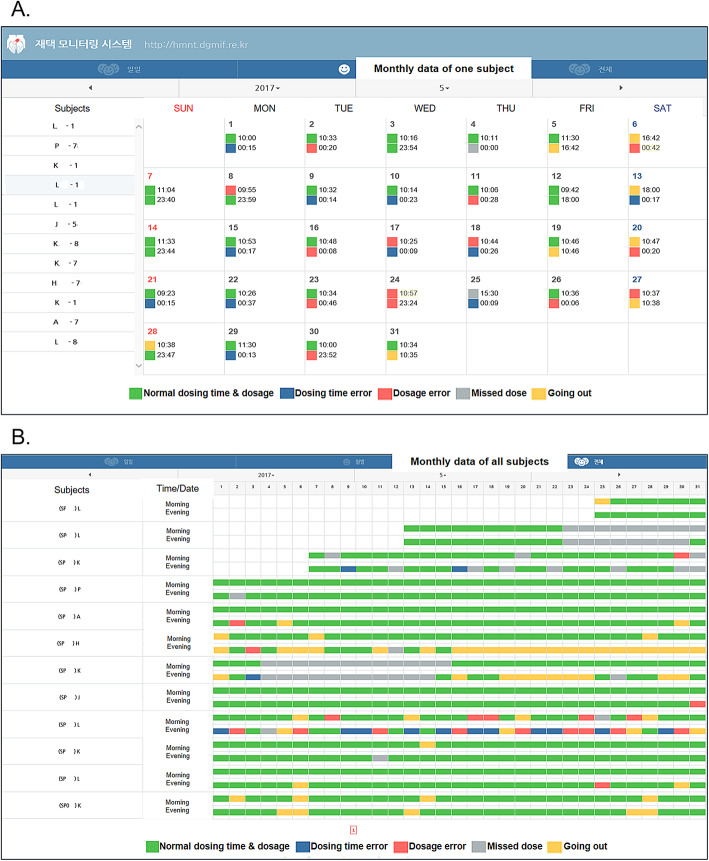
An example of adherence data in the intervention group as presented in the electronic case report form system. **a** Monthly data for one subject. **b** Monthly data for all subjects

In the intervention group, a total of 25 significant alarms and feedback messages were generated for 13 KTRs: 17 for missed doses, 6 for dosage errors, and 2 for dosing time errors. The following measurements in the intervention group did not significantly differ according to the number of feedback messages generated: tacrolimus trough levels (5.1 ± 1.2 vs. 5.3 ± 1.1, *p* = 0.574) tacrolimus CV (29.4 ± 16.3 vs. 22.1 ± 12.0, *p* = 0.155), mycophenolic acid trough levels (2.4 ± 1.0 vs. 2.9 ± 1.8, *p* = 0.332), mycophenolic acid CV (36.6 ± 24.8 vs. 38.3 ± 14.3, *p* = 0.754), renal allograft function at 4 weeks (71.5 ± 21.9 vs. 66.6 ± 16.9, *p* = 0.474) and at 24 weeks (65.7 ± 23.5 vs. 65.0 ± 17.4, 0.928), and the incidence of development of de novo anti-HLA antibodies (7.7% vs 5.3%, *p* = 0.555) (Table 3). Figure 3 shows an example of adherence data in the intervention group as presented in the electronic case report form system. This report allows medical staff to check on the patient’s medication use, dosing time, and dosage.

**Table 3:** Transplant outcomes of the intervention group according to the number of feedback messages generated

	Feedback ≥1 (*n* = 13)	No feedback (*n* = 38)	*p* value
Drug levels			
TAC trough level, ng/mL	5.1 ± 1.2	5.3 ± 1.1	0.574
TAC CV^a^	29.4 ± 16.3	22.1 ± 12.0	0.155
MPA trough level, μg/mL	2.4 ± 1.0	2.9 ± 1.8	0.332
MPA CV^a^	36.6 ± 24.8	38.3 ± 14.3	0.754
eGFR			
4 weeks	71.5 ± 21.9	66.6 ± 16.9	0.474
8 weeks	69.8 ± 22.7	67.3 ± 18.8	0.720
12 weeks	66.4 ± 21.0	66.8 ± 19.1	0.946
16 weeks	66.6 ± 19.4	67.9 ± 16.9	0.829
20 weeks	66.9 ± 22.2	66.1 ± 16.6	0.914
24 weeks	65.7 ± 23.5	65.0 ± 17.4	0.928
Number of events, *n* (%)			
De novo anti-HLA antibodies	1 (7.7)	2 (5.3)	0.748
BK viremia	0 (0)	1 (2.6)	0.555
BPAR	–	–	
DCGL	–	–	

Values are shown as mean ± standard deviation or number (%)

*BPAR* Biopsy-proven acute rejection, *CV* Coefficient of variation, *DCGL* Death-censored graft loss, *eGFR* estimated glomerular filtration rate, *HLA* Human leukocyte antigen, *MPA* Mycophenolic acid, *TAC* Tacrolimus

^a^CV = (standard deviation/mean) × 100%

### System satisfaction

Table 4 shows the general information regarding patients who completed the ICT-based clinical trial system satisfaction questionnaire. Of these patients, 50.0% were in their 50s, 57.1% were men, and 76.2% lived in large cities. All patients used a smartphone, and they searched for health information (information about symptoms, medications, etc.) on the Internet or through wireless communications with a mean frequency of 1.8 times per week.

**Table 4:** General information about patients who completed the ICT-based clinical trial system satisfaction questionnaire

Age, *n* (%)	
20s	2 (4.8)
30s	2 (4.8)
40s	9 (21.4)
50s	21 (50.0)
60s or above	8 (19.1)
Male, *n* (%)	24 (57.1)
Education level, *n* (%)	
Elementary school	3 (7.1)
Middle school	6 (14.3)
High school	23 (54.8)
University	9 (21.4)
Above university	1 (2.4)
Area of residence, *n* (%)	
Large city (metropolitan city)	32 (76.2)
Small- to medium-sized city	6 (14.3)
Agricultural and fishing village	4 (9.2)
Smartphone use, *n* (%)	42 (100)
Weekly frequency of searching health information (symptoms, medications, etc.) on the Internet or though wireless communications	1.8 ± 1.7
Occupation, *n* (%)	
Self-employment	11 (26.2)
Employee	7 (16.7)
Agricultural and livestock industry workers	2 (4.8)
Monk or Pastor	1 (2.4)
Student	1 (2.4)
Housewife	11 (26.2)
Not employed	9 (21.4)

Values are shown as mean ± standard deviation or number (%)

Table 5 shows the patients’ satisfaction with the ICT-based clinical trial system. The overall satisfaction with the system was above the median score, and significantly increased across the study period. The patients gave generally positive assessments of the system’s convenience, safety, and accuracy, and generally responded positively to the idea of using the system to participate in future clinical trials.

**Table 5:** ICT-based clinical trial system satisfaction questionnaire scores

	Visit 2	Visit 7	*p* value
Are you satisfied with the system, overall?	3.6 ± 1.0	3.9 ± 0.7	0.012
Was the system convenient to use?	3.6 ± 0.9	3.8 ± 0.8	0.294
Was it safe to use the system in the clinical trial?	4.1 ± 0.4	4.1 ± 0.7	0.767
Did use of the system reduce the duration of the trips made to participate in this clinical trial?	3.4 ± 0.8	3.4 ± 0.8	0.499
Did use of the system reduce the inconvenience associated with transportation?	3.3 ± 0.8	3.4 ± 0.8	0.618
Were the researchers able to more accurately assess your condition by using the system?	4.0 ± 0.7	3.9 ± 0.7	0.710
Did the devices included in the system (fingerprint sensor, home monitoring devices, gateway/smartphone apps, modem, etc.) provide reliable measurements?	3.8 ± 0.8	3.9 ± 0.7	0.844
Were the aforementioned devices easy to use?	3.9 ± 0.9	3.9 ± 0.8	0.872
Are you satisfied with the education regarding directions and precautions for using the aforementioned devices?	4.2 ± 0.7	4.1 ± 0.7	0.183
Are you satisfied with how the researchers handled errors that arose from the aforementioned devices?	4.3 ± 0.8	4.3 ± 0.8	0.200
Total scores	38.2 ± 5.8	38.8 ± 5.5	0.622
If the ICT-based centralized monitoring system is introduced into this clinical trial,			
Will you consistently participate in this clinical trial using the ICT-based centralized monitoring system?	3.9 ± 1.0	3.7 ± 0.8	0.323
Will you participate in this clinical trial even if it takes place at a hospital located farther away from your home owing to the availability of the system at that location?	3.3 ± 1.2	3.1 ± 1.0	0.648
Was this clinical trial using the system helpful for the management of your health?	3.9 ± 0.9	3.9 ± 0.9	0.578
Will this clinical trial using the system positively contribute to your quality of life?	3.7 ± 1.0	3.7 ± 0.8	0.660
Would you recommend a clinical trial using this system to others?	3.6 ± 1.1	3.8 ± 0.8	0.263
Do you think clinical trials using the system may lead to any losses or damage associated with personal medical information leakage?	3.5 ± 1.0	3.9 ± 0.9	0.054
Do you think it will become more difficult to use medical services owing to technical issues associated with the system?	3.7 ± 1.0	3.8 ± 0.8	0.893
Do you think technical issues associated with the system will give rise to medical accidents?	4.1 ± 0.8	3.9 ± 0.9	0.130
Total scores	29.6 ± 5.4	29.8 ± 4.2	0.932

Values are shown as mean ± standard deviation

Each domain is rated on a scale from 1 to 5, with higher scores reflecting better satisfaction

## Discussion

In this randomized clinical trial, we found that Korean KTRs already showed high adherence in terms of dosing and timing. The ICT-based centralized monitoring system did not significantly improve adherence to immunosuppressive medications or transplant outcomes in this population. However, the KTRs expressed overall high satisfaction with the ICT-based centralized monitoring system, and this satisfaction significantly improved across the study period. Participants gave an overall positive assessment of the system’s convenience, safety, and accuracy. Although this system did not maximize mediation adherence or enhance transplant outcomes in KTRs due to the already high baseline adherence, the high satisfaction indicates that the system could be successfully applied in future clinical trials targeting other disease groups with impaired adherence.

Previous studies have assessed the effects of technology-based adherence-promoting interventions—including the use of electronically monitored drug-dispensing devices or mobile phone applications with feedback (including alarms, text messages, telephone calls, e-mails, or face-to-face meeting)—and have demonstrated that such interventions are associated with higher adherence among KTRs or other organ transplant recipients [7–9, 13–16]. Table 6 summarizes recent studies evaluating technology-based adherence-promoting interventions in KTRs. Henriksson et al. tested the use of an electronic medication dispenser for 1 year to improve adherence among KTRs (40 intervention patients, 40 control patients), and reported that the intervention was associated with high adherence [9]. However, unlike our current study, that prior study did not measure adherence in the control group, and did not determine different aspects of adherence, such as dose-taking adherence, dose-frequency adherence, and dose-interval adherence. Reese et al. examined the use of electronic medication monitoring and reminders (including alarms, texts, telephone calls, and/or e-mails) either alone or in combination with provider notification for 6 months among KTRs (40 patients with reminders, 39 with reminders plus provider notification, and 38 control patients), and found that the intervention resulted in significantly better medication dose-taking adherence [8]. While our current study used only text messages and pill box alarms, Reese et al. may have made participants more comfortable with the system by enabling them to choose from customized reminders. In the study of Foster et al., KTRs (81 intervention patients, 88 control patients) received reminders (including text messages, e-mails, and/or visual cues for dose reminders) and had face-to-face meetings with a coach at 3-month intervals over 12 months. Their results demonstrated that the intervention led to significantly better medication dose-taking adherence and dose-frequency adherence [7]. Unlike our present study, that previous study included face-to-face feedback from a coach, which might contribute to different results. On limitation that is shared between previous studies and our present study is that the electronic pill bottles and the smart pill box only measure opening, but do not confirm actual pill ingestion.

**Table 6:** Studies evaluating technology-based adherence-promoting interventions among kidney transplant recipients

Authors, Year [Ref.]	Study design and sample	Intervention	Duration	Adherence	Results for adherence and clinical outcomes	Advantage	Disadvantages
Henriksson et al., 2016 [9]	RCT *n* = 80 (40 intervention, 40 control)	Device: Electronic medication dispenser Feedback: emitted visual and audible alerts	12 months	Dose-taking adherence	No significant differences in tacrolimus trough levels, risk of BPAR, or creatinine levels		No adherence information in the control group Measured only dispenser opening, not actual pill ingestion
Reese et al., 2017 [8]	RCT *n* = 117 (40 reminders, 39 reminders plus provider notification, 38 control)	Device: Electronic medication monitor and reminders either alone or in combination with provider notification Feedback: alarms, texts, telephone calls, and/or e-mails	6 months	Dose-taking adherence	Significantly better adherence with reminders plus provider notification and with reminders alone compared to in the control group No significant difference in tacrolimus trough levels	Various feedback methods	Measured only dispenser opening, not actual pill ingestion
Foster et al., 2018 [7]	RCT *N* = 169 (81 intervention, 88 control)	Device: Electronic medication monitor and face-to-face education Feedback: text messages, e-mails, and/or visual cue dose reminders	12 months	Dose-taking adherence and dose-frequency adherence	Intervention group had significantly better adherence than the control group No significant difference in the standard deviation of tacrolimus trough levels	Various feedback methods	Measured only dispenser opening, not actual pill ingestion
Jung et al., 2020 [the current study]	RCT *N* = 105 (51 intervention, 54 control)	Device: Smart pill box Feedback: text messages, pill box alarms	6 months	Dose-taking adherence, dose-frequency adherence, and dose-interval adherence	No significant difference in adherence, tacrolimus and mycophenolic acid trough levels, coefficient of variation of drug levels, and risk of the development of de novo anti-HLA antibodies	The ICT-based centralized monitoring system can be linked to not only smart pill box but also blood sugar meter, electrocardiogram, spirometry, and INR meter	Measured only box opening, not actual pill ingestion

*HLA* Human leukocyte antigen, *ICT* Information and communication technology, *RCT* Randomized controlled trial

With regards to the effects of interventions on transplant outcomes, previous studies have reported conflicting results. We hypothesized that the ICT-based centralized monitoring system could improve medication adherence in KTRs, ultimately inducing stable drug concentrations and thus reducing the development of de novo anti-HLA antibodies, viral infection, and BPAR. Contrary to our expectations, drug adherence, tacrolimus and MPA trough concentrations, drug level variability, and the incidences of development of de novo anti-HLA antibodies, BK viremia, and BPAR did not significantly differ between the ICT-based centralized monitoring group and the control group, or within the intervention group between patients with versus without feedback generation. Compared with previous studies, the KTRs in our present study exhibited considerably higher adherence of 99–100%. Therefore, there was little-to-no room for improvement based on feedback generated from the ICT-based centralized monitoring system. Consequently, we did not observe superior transplant outcomes in the ICT-based centralized monitoring group compared with the control group, or in the patients with versus without feedback generation within the intervention group.

Although we did not find that the monitoring system led to improved adherence due to the already high baseline adherence in Korean KTRs, this study has several strengths and the results have clinical implications. First, to our knowledge, this is the first study to investigate the adherence of Korean KTRs with continuous use of an ICT-based centralized monitoring system. Moreover, it is the first clinical study to provide data regarding medication adherence among Korean KTRs. The higher adherence observed in Korean KTRs compared to in KTRs in other countries could be partly due to the national health insurance coverage system for transplant recipients in South Korea. Out present data suggest that the proportion of non-adherence leading to antibody-mediated rejection may differ among countries [1], and that the causes of renal allograft loss may also differ. This indicates that different and additional immunological strategies may be required to improve renal allograft survival in different countries. Second, the overall satisfaction with the system was higher than neutral, even though most users were in their 50s or older. This suggests that the ICT-based centralized monitoring system could be applied to other diseases, such as recipients of other organ transplants, or patients with hypertension, diabetes, chronic kidney disease, cardiovascular disease, human immunodeficiency virus infection, dementia, and tuberculosis. Moreover, the high satisfaction with the ICT-based clinical trial could pave the way for establishing an ICT-based centralized monitoring system for future clinical trials. Further system validation will be necessary to expand the use of this monitoring system in future clinical trials.

## Conclusions

In conclusion, in our present study, the ICT-based centralized monitoring system did not improve mediation adherence or transplant outcomes in Korean KTRs due to the already high baseline adherence. However, we found high patient satisfaction with the system in terms of convenience, safety, and accuracy. This suggests that the ICT-based centralized monitoring system could be successfully used in future clinical trials targeting other disease groups with impaired adherence and in which medication adherence critical to the course of treatment.

### Advantages and disadvantages

Through this clinical trial, we identified the following advantages and disadvantages of the ICT-based centralized monitoring system. First, this system enabled us to receive real-time information on adherence collected through the smart pill box, check this information using an electronic case report form system, and provide patients with real-time feedback regarding dose, frequency, and interval. This enabled medical staff to identify patients’ patterns and exact times of taking medicines, and helped them understand the levels of immunosuppressive agents in the outpatient clinic. Second, the ICT-based centralized monitoring system could be linked to not only the smart pill box but also to a blood sugar meter, electrocardiogram, spirometry, and INR meter. Therefore, a strength of this system is that it can be successfully used in other diseases that require constant monitoring due to severe fluctuations in symptoms or results. Third, like recent studies that have introduced systems that collect current information and predict future trends [17–20], once our adherence-related information is accumulated, it will also be available to predict future patterns of adherence in KTRs. Forth, some patients experienced technical problems, such as failed fingerprint recognition or incorrect reminders while they were out. However, these technical issues were all resolved early in the study. Fifth, in areas with inconsistent Internet communication, there may be problems with data transmission.

## Supplementary information

**Additional file 1.**

## Data Availability

Data supporting the findings of the current study are available from the corresponding author upon reasonable request.

## Abbreviations

BPAR: Biopsy-proven acute rejection
CV: Coefficient of variation
DCGL: Death-censored graft loss
eGFR: estimated glomerular filtration rate
ICT: Information and Communication Technology
KTRs: Kidney transplant recipients
PRA: Panel-reactive antibody
RCT: Randomized controlled trial
